# Advances in Preoperative and Intraoperative Technologies for Safe Resection of Gliomas in Cognitive Regions

**DOI:** 10.3390/cancers17243890

**Published:** 2025-12-05

**Authors:** Valentina Vintimilla Rivadeneira, Jose E. Leon-Rojas

**Affiliations:** 1 NeurALL Research Group, Quito 170157, Ecuador; valentina.vintimilla@udla.edu.ec; 2 Cerebro, Emoción y Conducta (CEC) Research Group, Escuela de Medicina, Universidad de las Américas (UDLA), Quito 170124, Ecuador

**Keywords:** glioma surgery, functional mapping, awake craniotomy, connectomics, fluorescence-guided surgery

## Abstract

Advances in neuroimaging and intraoperative mapping have transformed brain tumour surgery from anatomy-based resection to function-guided intervention. This review synthesises current evidence on multimodal strategies for maximising tumour removal while preserving cognitive and neurological function. Integrating task-based and resting-state functional MRI (fMRI), diffusion tensor imaging (DTI), tractography, and connectomic analysis enables personalised mapping of eloquent and cognitive networks. Intraoperatively, awake craniotomy with direct electrical stimulation (DES) remains the gold standard for real-time functional validation, while adjuncts such as intraoperative MRI (iMRI), 5-aminolevulinic acid (5-ALA) fluorescence, and ultrasound-based extended resection accuracy. However, these technologies present unique limitations, including neurovascular uncoupling in fMRI, tract distortion in DTI, and resource constraints in low-income settings. Our review differentiates their application across low-grade and high-grade gliomas, emphasising that tumour biology determines the balance between neuroplasticity-driven mapping and imaging-guided radicality. Key future priorities include validation of multimodal imaging protocols, integration of longitudinal neuropsychological outcomes, and development of interpretable connectomic models. Addressing the technological and ethical challenges of high-field MRI, data standardisation, and cost-effective implementation will be essential for equitable global adoption. Ultimately, the evolution of functional neurosurgery depends not only on new technologies but on integrating multimodal evidence and patient-centred outcome measures to achieve reproducible, safe, and personalised brain tumour surgery.

## Simple Summary

Brain surgery for tumours has evolved from simply removing as much tissue as possible to protecting the functions that make each person unique, such as speech, memory, and movement. Our review explains how new imaging and mapping techniques now allow surgeons to see which areas of the brain are essential before and during an operation. Using tools like advanced MRI scans, real-time imaging, and awake procedures, doctors can remove more of the tumour while keeping important abilities intact. Our article also discusses how these technologies can be adapted to different hospital settings and what challenges remain, including cost, accessibility, and the need for standard methods. By bringing together knowledge from imaging, surgery, and neuroscience, this research aims to help develop safer, more precise, and more personalised approaches to brain tumour treatment worldwide.

## 1. Introduction

Gliomas are the most common primary malignant brain tumours in adults, accounting for approximately 81% of all malignant brain neoplasms and 22.9% of all tumours [1]. They range from slow-growing low-grade gliomas (LGGs, WHO grade 2) to aggressive high-grade gliomas (HGGs), including glioblastomas (GBM, WHO grade 4), which remains one of the most lethal cancers despite multimodal treatment [2,3]. Across this spectrum, the extent of surgical resection remains one of the most relevant predictors of overall survival and progression-free survival [4,5]. Numerous studies have confirmed that maximal resection is associated with delayed tumour progression, improved seizure control, and increased responsiveness to adjuvant therapies such as chemotherapy and radiotherapy [4,5]. However, the drive to achieve maximal resection must be tempered by the risk of damaging critical brain regions responsible for essential functions. Historically, neurosurgical decision-making was guided by a relatively coarse anatomical classification into “eloquent” and “non-eloquent” regions, based largely on motor and language maps [6,7]. While avoiding motor deficits and aphasia is still an important endeavour, emerging evidence suggests that higher-order cognitive functions, including memory, attention, executive functioning, and visuospatial abilities, are equally or even more essential for quality of life and social reintegration after surgery [8,9,10]; for instance, a radio presenter will find losing his speech ability (due to a lesion in Broca’s or Wernicke’s area) disastrous, but it would be just as disastrous to have a lesion in the non-dominant hemisphere that will result in the loss of prosody (voice intonation and conveying emotion). Cognitive impairment following glioma surgery can be as disabling as focal neurological deficits, affecting the patients’ independence, employability, and psychosocial well-being [8,9,10]. Consequently, neurosurgical oncology has shifted toward an onco-functional balance, meaning that the goal is no longer merely maximal resection, but maximal safe resection while preserving the intricate neural networks that underlie cognition and other relevant functions [9]. In this review, the term cognitive regions refers to large-scale, higher-order brain networks that sustain complex processes such as attention, working memory, executive control, language, etc. The default mode network (DMN), encompassing the medial prefrontal cortex, posterior cingulate cortex, and angular gyrus, supports introspection and memory retrieval and is often disrupted by tumours infiltrating medial parietal or frontal areas. The executive control network (ECN), involving the dorsolateral prefrontal and posterior parietal cortices, regulates goal-directed behaviour and cognitive flexibility and frequently demonstrates compensatory reorganisation, particularly in patients with frontal or insular gliomas. The salience network (SN), anchored in the anterior insula and dorsal anterior cingulate cortex, orchestrates dynamic switching between the DMN and ECN to prioritise behaviourally relevant stimuli, while the frontoparietal and attention networks contribute to attentional control and working memory [8,9,10]. These interconnected systems underscore that cognitive alterations in brain tumour patients often arise from network-level disruptions rather than focal cortical injury alone.

This paradigm shift has been made possible by technological advances in both preoperative planning and intraoperative monitoring. Preoperatively, techniques such as functional magnetic resonance imaging (fMRI), both task-based and resting-state, along with diffusion tensor imaging (DTI) and tractography, allow clinicians to non-invasively delineate cortical functional regions and their subcortical white matter connections [11,12]. This functional mapping facilitates a complex and thorough understanding of individual patients’ brain organisation, especially considering that gliomas often induce functional reorganisation and plasticity. Intraoperatively, awake craniotomy combined with direct electrical stimulation (DES) enables real-time mapping of not only motor and language functions but also cognitive domains, allowing surgeons to continuously test patients during resection [13]. Adjunct tools such as fluorescence-guided surgery (FGS) with agents like 5-aminolevulinic acid (5-ALA) and intraoperative imaging (MRI, ultrasound, CT) can provide real-time visualisation of tumour margins and residual disease, thus maximising tumour removal without transgressing functional boundaries [14]. Moreover, the field is moving toward connectome-based neurosurgery, in which the brain is not viewed as a series of isolated functional regions but as a dynamic network of interconnected nodes (i.e., a connectionism approach) [15]. This perspective is particularly pertinent for cognitive functions, which depend on the integrity of distributed networks rather than single loci [16,17]. As a result, preserving connectivity, particularly in association fibres such as the arcuate fasciculus, inferior fronto-occipital fasciculus, and cingulum, is becoming a central concern in glioma surgery [16,17]. Connectomic approaches integrate functional and structural imaging data to generate patient-specific models of brain organisation, enabling a level of surgical individualisation that was previously unthinkable.

We conducted a comprehensive literature search to identify studies investigating functional connectome alterations and its relationship with brain tumours. The databases PubMed/MEDLINE, Scopus, Web of Science Core Collection, and the Virtual Health Library (VHL) were systematically searched for articles published from inception to June 2025. Reference lists of eligible studies and relevant reviews were also screened manually to identify additional publications. The search strategy combined controlled vocabulary (MeSH and DeCS terms) with free-text keywords, using Boolean operators such as (“brain tumor” OR “glioma” OR “astrocytoma” OR “brain neoplasm”) AND (“functional connectivity” OR “connectome” OR “functional MRI” OR “resting-state fMRI” OR “tractography” OR “DTI”) AND (“surgery” OR “preoperative planning” OR “intraoperative” OR “surgical techniques” OR “multimodal imaging”), with equivalent syntax applied across databases. Studies were included if they were original human research articles published in peer-reviewed journals, investigated primary or metastatic brain tumours, and incorporated functional or structural neuroimaging methods such as fMRI, DTI, or tractography to evaluate connectomic changes and their utility in pre-operative planning or intra-operative execution. Exclusion criteria comprised case reports with fewer than three patients, conference abstracts, editorials, studies without neuroimaging data, and preclinical or in vitro models. Two reviewers independently screened titles, abstracts, and full texts, resolving disagreements by consensus. Data were extracted into a structured template encompassing study design, tumour type, imaging modality, and principal findings.

Our narrative review aims to synthesise recent advances in preoperative neuroimaging and intraoperative techniques that have transformed the surgical management of adult gliomas affecting cognitive domains. We will focus on the integration of functional MRI, tractography, intraoperative mapping, and real-time imaging, as well as their role in facilitating awake cognitive mapping and the emerging field of network-based surgical strategies. We will also address current limitations, technological challenges, and future directions; by adopting a multimodal, function-preserving approach, contemporary neurosurgery is well equipped to prolong survival while preserving the mind.

## 2. Preoperative Functional Mapping and Connectomics

**Figure 1:**
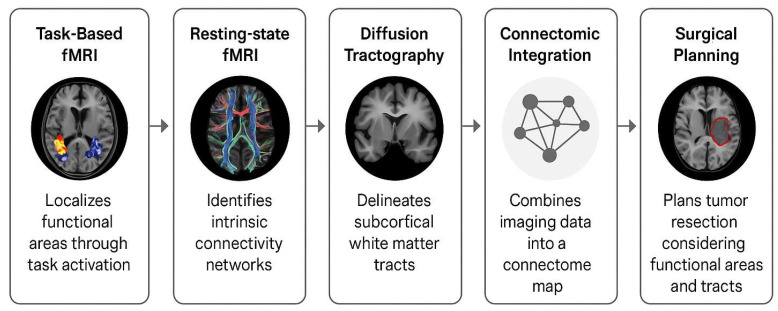
Multimodal Preoperative Workflow for Connectome-Based Glioma Surgery.

Preoperative planning is critical for tumours in or near the cortex or subcortical pathways. Modern neuroimaging can noninvasively localize key functional regions and networks, informing surgical approach and risk assessment. Figure 1 shows a basic preoperative assessment workflow using multimodal imaging.

### 2.1. Task-Based fMRI for Functional Localization

Functional MRI (fMRI) is frequently used to map eloquent and functional cortical areas preoperatively; task-based fMRI (tb-fMRI) involves having the patient perform specific tasks (e.g., language tasks such as word generation, semantic decision, or motor tasks like finger tapping) during MRI scanning to visualize task-evoked activation [18]. Tb-fMRI can localize critical regions such as Broca’s and Wernicke’s areas for language or the motor cortex for movement, and their location relative to the tumour [18,19]. Numerous studies have shown that tb-fMRI has good concordance with intraoperative mapping and can reduce surgical morbidity [11]. For example, a 2021 meta-analysis of 68 studies (3280 patients) found that patients who underwent preoperative fMRI had significantly lower odds of postoperative neurological deficits (approximately one-fourth the risk) compared to those without fMRI mapping [18]. The pooled rate of new postoperative deficits was approximately 11% with fMRI versus 21% without, and functional outcomes (e.g., Karnofsky performance status) were better in the fMRI-planned group [18]. These findings suggest that incorporating fMRI into surgical planning can help surgeons avoid critical areas, thereby reducing postsurgical morbidity. As a result, clinical fMRI is now an established tool in brain tumour surgery, often used in combination with diffusion tractography to map cortico-subcortical networks.

Despite its utility, tb-fMRI has limitations. It requires patient cooperation and task performance, which may be challenging if patients have neurologic deficits or are anxious [20]. Moreover, task design is crucial; language mapping, for instance, may need multiple paradigms (e.g., verbal fluency, picture naming, reading) to cover the full network. Even then, false negatives can occur if a patient’s particular functional organization or task strategy does not activate all critical regions, or if tumour-related neuroplasticity has shifted functions [20,21]. Additionally, tb-fMRI can produce false positives by activating regions that are not strictly essential (for example, task-related but noncritical regions or “network-adjacent” areas) [20]. To mitigate this, thresholding and careful interpretation by experienced neuro-radiologists and neurosurgeons is required.

### 2.2. Resting-State fMRI and Network Mapping

**Figure 2:**
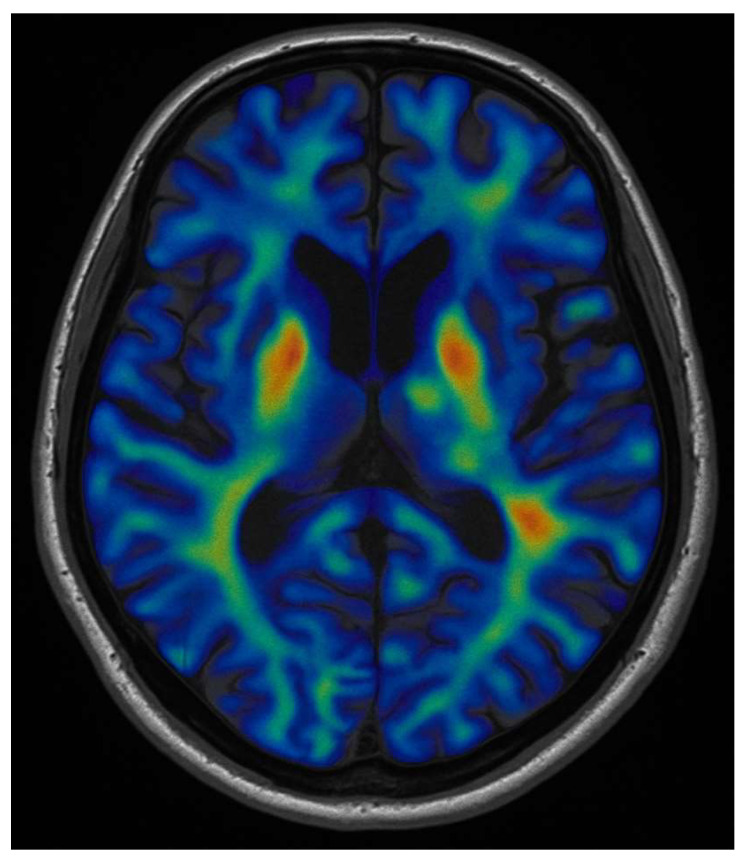
Axial resting-state fMRI showcasing spontaneous neural activity, typically measured as blood-oxygen-level-dependent (BOLD) signal fluctuations during rest. The coloured overlay (ranging from blue to red) corresponds to the strength of correlated activity between brain regions, representing the Default Mode Network (DMN). Areas highlighted in orange represent activation of the ventral pallidum, an important subcortical structure involved in this network.

In contrast to tb-fMRI, resting-state fMRI (rs-fMRI) has been increasingly used to map functional networks without requiring active tasks. Rs-fMRI measures spontaneous low-frequency fluctuations in BOLD signal while the patient lies at rest, and through correlation analysis, identifies coherent networks (e.g., the default mode network, as shown in Figure 2; the sensorimotor network, or the language network) [19,22].

Recent work has shown that rs-fMRI can localize language networks in brain tumour patients with reliability approaching that of task-based methods; in fact, rs-fMRI may reveal more extensive network connectivity than task activation, since it is not limited to task-specific pathways [19,22,23]. For patients unable to perform tasks (due to young age, cognitive impairment, or language barriers), rs-fMRI offers a non-invasive alternative for presurgical mapping of language and other cognitive networks. Park et al. (2020) directly compared task-based and resting-state fMRI for language mapping in 35 patients and found that both methods identified key language areas (Broca’s and Wernicke’s) with overlap [19]. Interestingly, their analysis also showed that task-fMRI activated some domain-general regions (e.g., working memory areas) beyond core language areas, whereas classifier-based rs-fMRI produced a more specific map of the language network confined to language-specific regions [19]. This suggests rs-fMRI may avoid some of the nonessential activations occurring in task paradigms, providing a focused view of intrinsic language networks.

Clinically, rs-fMRI is gaining acceptance as part of the preoperative workup. Advanced analytical techniques (independent component analysis, machine learning classifiers, etc.) have improved the extraction of networks from resting data in individual patients [22]. A recent feasibility study in tumour patients concluded that rs-fMRI can replace standard task-fMRI for language mapping in routine practice, as it identified Broca’s and Wernicke’s areas in all cases and additionally showed connectivity between them [24]. Importantly, rs-fMRI does not require patient participation in tasks, making it applicable in a broader range of patients. As processing pipelines become more automated and user-friendly, rs-fMRI network mapping could complement or, in some cases, substitute task-based mapping [19,24]. Both approaches together move neurosurgery toward a connectomic perspective, mapping not just isolated cortical regions, but the larger-scale networks and interconnections that support cognitive functions.

### 2.3. Diffusion Tensor Imaging and Tractography

While fMRI (tb-fMRI and rs-fMRI) localizes cortical functional areas, diffusion tensor imaging (DTI) provides a map of subcortical white matter pathways that must be preserved to maintain function. DTI uses diffusion-weighted MRI to model the orientation of water diffusion in brain tissue, allowing reconstruction of major fibre tracts via tractography [25]. In the context of glioma surgery, DTI tractography is commonly employed to visualize important functional tracts like the corticospinal tract (motor pathway), arcuate fasciculus (language pathway), inferior fronto-occipital fasciculus and inferior longitudinal fasciculus (language/semantic and visual association pathways), optic radiations (vision), cingulum (memory network), and others that run near the lesion [26,27,28]. Knowledge of a tumour’s relationship to these fibres (displaced, infiltrating, or intersecting them) critically informs surgical risk [29]. For example, tractography of the arcuate fasciculus (connecting frontal and temporal language areas) can predict the risk of postoperative aphasia; if the tumour encases or disrupts the tract, aggressive resection carries a high likelihood of permanent language deficit [26,30]. In one study of insular/perisylvian gliomas, patients with intact preoperative language tracts on DTI had significantly better language outcomes after surgery than those with tumour involvement of these tracts [28]. Such data showcase that tract integrity is a key limiting factor in functional recovery of gliomas affecting cognitive areas, and DTI helps stratify patients’ risk and plan extent of resection accordingly.

Beyond risk prediction, DTI fibre tracking is now routinely integrated into neuronavigation systems for surgical guidance. The tractography can be imported into the surgical navigation MRI, allowing the surgeon to see the 3D course of, for instance, the optic radiations relative to the tumour in real time [31]. This guides intraoperative decisions on where to stop resection to avoid transecting a critical pathway. DTI has enabled mapping of subcortical language pathways (such as the superior longitudinal fasciculus, frontal aslant tract, etc.) which historically were difficult to localize even with cortical mapping techniques [32]. However, intraoperative stimulation remains the gold standard for identifying functional white matter, but DTI provides a valuable preoperative blueprint and can reduce mapping time by narrowing the search to specific tract regions [33]. In a meta-analysis of presurgical mapping, the greatest benefits in outcome were seen when fMRI was combined with DTI tractography and intraoperative stimulation, highlighting that a multimodal approach yields the best functional results [18].

However, DTI is not perfect; the technique may have reduced accuracy in areas of complex crossing fibres or in regions distorted by tumour and oedema [34]. False continuations or premature terminations of tracts can occur due to diffusion signal noise or algorithm limitations. Advanced diffusion models (high angular resolution diffusion imaging (HARDI), tractography with free-water correction, constrained spherical deconvolution or neurite orientation dispersion and density imaging (NODDI)) are emerging to improve tract delineation in the presence of such adverse conditions that can distort normal anatomy [34,35,36]. Researchers are also exploring connectomic analyses that integrate DTI and functional data, creating patient-specific network models [37]. Overall, DTI tractography has become an important component of glioma surgery planning, allowing surgeons to approach the tumour with an understanding of the subcortical pathways that must be respected.

Table 1 provides a summary comparison of the discussed pre-operative technics assessing their main likely and impact in surgical decision-making.

### 2.4. Connectomic Planning and Predictive Models

The convergence of functional and diffusion imaging has given rise to a connectomic approach in neurosurgery. Instead of treating a brain tumour in isolation, surgeons now consider the patient’s unique brain network architecture, identifying which networks are affected by the tumour and which are critical to preserve for quality of life, including those in the wrongly called “non-eloquent” areas [38,39]. Connectomic analysis can involve mapping functional hubs and network nodes (from fMRI) and the connecting fibre bundles (from DTI), yielding a holistic map of brain connectivity around the tumour. This approach aligns with the conceptual shift described by Duffau (2017) as moving from “image-guided surgery” to “functional mapping–guided surgery” [38]. In practical terms, connectomic planning might mean delineating not just primary motor cortex, but the entire motor network (premotor, supplemental motor, basal ganglia, corticospinal tract); not just Broca’s area, but the distributed language network (inferior frontal, superior temporal, parietal regions, and the connecting arcuate and semantic tracts) [15,39,40]. By understanding the network context of a tumour, the surgeon can tailor resection boundaries to spare crucial cognitive network nodes or minimize disconnection of hubs that would cause cognitive disability. An example of connectome-based planning is the use of resting-state fMRI network maps to guide glioma surgery; resting-state connectivity may identify atypical organization (e.g., right-hemisphere language lateralization or recruitment of auxiliary networks) that would not be evident from anatomy alone [41]. Similarly, structural connectome analysis might reveal an alternate pathway that could compensate for a resected one (leveraging neuroplasticity), thus influencing surgical strategy. New predictive models are being developed that combine tumour location with connectomic features to estimate the probability of various deficits. For instance, a tractography-based risk model for language outcome can quantify how much infiltration or distance from language tracts correlates with postoperative aphasia risk [40]. Such models help in patient counselling and surgical decision-making (e.g., whether to attempt gross total resection or stop at subtotal to preserve function).

Advanced preoperative imaging, including task and resting fMRI, DTI tractography, and connectomic analytics can currently provide a comprehensive map of the brain’s functional landscape [39]. This map forms the foundation of surgical planning aimed at maximal safe resection. The ultimate goal is not only to remove what is visible on an MRI, but to resect tumour to the limits of functional brain boundaries, always respecting functionality and patient-tailored quality of life [39]. Preoperative mapping can significantly reduce the risk of surgery as it allows patient-specific tailoring, sometimes even opting for neoadjuvant therapies if mapping suggests unacceptable risk, and it sets the stage for employing intraoperative adjuncts to further protect function and reduce the surgical footprint in the brain.

**Figure 3:**
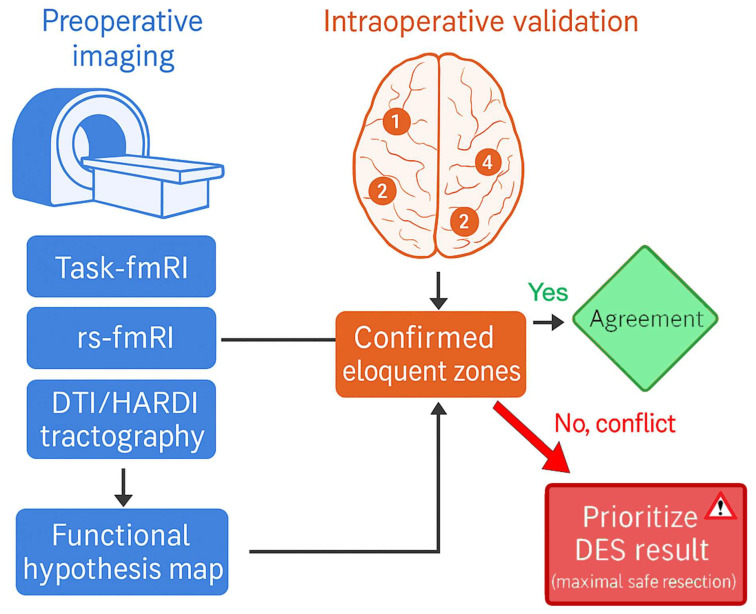
Hierarchical workflow for multimodal functional decision-making in brain tumour surgery. Schematic representation of the integrative approach combining preoperative imaging and intraoperative validation for functional mapping. Preoperative imaging modalities (task-based fMRI, resting-state fMRI, and DTI/HARDI tractography) are used to construct a functional hypothesis map that guides surgical planning. Intraoperative validation using direct electrical stimulation (DES) confirms eloquent cortical and subcortical regions. When findings from different modalities conflict, priority is given to DES results to ensure functional preservation. The final integrated surgical plan balances maximal tumour resection with maintenance of critical network integrity.

Although a multimodal neuroimaging approach provides a more comprehensive understanding of tumour-related brain reorganisation, discrepancies among modalities are not uncommon. For instance, functional MRI may indicate a non-functional cortical region due to neurovascular uncoupling, while direct electrical stimulation (DES) confirms preserved function [15,38,39,40,41,42,43]. In such cases, clinical decision-making must prioritise intraoperative functional validation, as DES remains the gold standard for determining cortical and subcortical eloquence [15,38,39,40,41,42,43]. Preoperative techniques such as task-based and resting-state fMRI, diffusion tractography, and advanced diffusion models serve as valuable guides for surgical planning, but their interpretations should be integrated within a hierarchical framework that accounts for each modality’s physiological assumptions and limitations (Figure 3). A principled approach involves using preoperative imaging to generate functional hypotheses, which are then confirmed or refined intraoperatively through DES and neurophysiological monitoring. This integration minimises the risk of false negatives from imaging-based modalities and supports maximal safe resection while preserving essential networks (Figure 3).

## 3. Intraoperative Technologies for Maximizing Safe Resection

Once in the operating room, a variety of intraoperative tools can be employed to ensure the resection is as complete as possible without causing neurological and cognitive injury. These include direct functional monitoring and mapping techniques as well as real-time imaging and fluorescent visualization of tumour tissue. The following sections detail recent advances in each domain.

### 3.1. Awake Craniotomy with Direct Electrical Stimulation

For tumours near critical cortical or subcortical areas, awake craniotomy with direct electrical stimulation (DES) mapping is considered the gold standard for maximizing resection safely [38,42]. In this technique, the patient is awakened (or kept awake) during key parts of the surgery to allow continuous neurological testing while the neurosurgeon applies brief electrical currents to areas of cortex or white matter before cutting them [42,43]. Positive mapping (eliciting a response or disruption) indicates the region is functional and should be preserved. Historically, intraoperative DES was used mainly for motor and speech mapping (e.g., stimulating the motor strip to induce movement or naming areas to induce speech arrest) [44]. Over the last decade, however, mapping has expanded to higher cognitive and behavioural functions in order to protect the full spectrum of patient abilities [38,45,46]. As survival has improved, especially for low-grade gliomas, preserving cognitive functioning and quality of life has become a top priority [42]. Merely avoiding paralysis or aphasia is no longer sufficient; neurosurgery now strives to prevent subtle but impactful deficits in memory, multitasking, social cognition, executive functioning, etc, that could derail a patient’s professional and personal life.

Cognitive mapping during awake surgery involves tailoring intraoperative tests to the tumour location and the patient’s needs [47]. For a tumour near language areas, surgeons map not only basic speech (counting, object naming) but also language semantics, syntax, reading, and even non-verbal language like prosody if relevant [48,49]. For frontal tumours, tasks assessing executive function (working memory, inhibition tasks), attention (continuous performance tasks), or social cognition (emotion recognition, theory-of-mind questions) may be introduced [48,49]. If a tumour is near parietal regions, tasks for visuospatial abilities (e.g., line bisection, spatial working memory) can be used [48,49]. One innovative approach is concurrent multi-tasking under time pressure during mapping, essentially mimicking real-life cognitive load [50]. Duffau and colleagues (2022) proposed that having the patient perform dual tasks (for example, a verbal task and a hand motor task simultaneously) with time constraints can reveal connectivity that might not be apparent when tasks are completed in isolation [50]. They observed cases where a patient could perform each task separately without issue, but stimulation of a certain site caused breakdown in performance when multitasking, indicating that site was critical for inter-network coordination [50]. This “meta-network” monitoring is an emerging strategy to ensure integrated cognitive functions (like divided attention or rapid task-switching) are preserved, beyond just single-task abilities [47,50,51].

During awake mapping, DES is applied at the cortical surface (typically 1–4 mA currents for 1–4 s) to transiently disrupt function, and at the subcortical level (often higher current 5–8 mA) as resection proceeds deeper [51]. The patient engages in the selected tasks continuously. If stimulation causes errors (e.g., speech arrest, incorrect responses, confusion, paraesthesia), the surgeon marks that location as “eloquent” and avoids resecting it. In contrast, tumour tissue usually does not elicit any deficit when stimulated, allowing safe removal [51]. Real-time monitoring of patient responses is crucial; a trained neuropsychologist or speech-language pathologist in the OR often administers and scores the tasks. Through DES mapping, the surgeon can tailor the resection margin on the fly, stopping at the point where further removal would cause a deficit. This technique has enabled resection of tumours that were once deemed inoperable, with impressive outcomes [33,42]. For low-grade gliomas, studies show that with awake mapping, >90% gross total resection rates are achievable in non-paralytic eloquent locations while permanent deficits are exceedingly low [13]. In a recent series, early and aggressive resections guided by DES in low-grade glioma patients have yielded median overall survival beyond 15–20 years with nearly no long-term neurological impairments: a remarkable improvement over historical outcomes [9].

For high-grade gliomas in eloquent areas, awake DES mapping likewise improves extent of resection and reduces deficits [33]. A meta-analysis found that glioblastoma surgeries conducted with brain mapping had significantly lower rates of severe neurologic deficits postoperatively (approximately 3.4%) compared to those without mapping (an estimated 8–29%) [42]. Awake mapping is now considered standard of care for tumours near speech or motor areas, and its use is expanding to other cognitive territories [13,49]. However, challenges remain: not all patients are candidates (some cannot tolerate awake surgery due to anxiety or medical conditions), and mapping of very complex functions (like memory encoding or abstract reasoning) is still limited by lack of rapid intraoperative tests and less clear localization of complex functions [47,49]. Suitable candidates are typically those with tumours located near eloquent cortical or subcortical regions who demonstrate adequate baseline cognitive function, motivation, and psychological resilience [42,47,49]. Preoperative neuropsychological evaluation is recommended to confirm that the patient can cooperate with intraoperative testing and tolerate transient functional disruptions during stimulation. Contraindications include severe anxiety or psychiatric disorders, poor language comprehension, significant hearing impairment, respiratory instability, or inability to remain motionless during the awake phase [47,49]. Additionally, children, patients with high intracranial pressure, or those requiring complex skull-base approaches may be better managed under general anaesthesia. For patients deemed ineligible, alternative intraoperative monitoring techniques such as asleep motor mapping with continuous electromyography and motor-evoked potentials offer partial functional assessment. In such cases, integration of preoperative functional MRI, DTI tractography, and neuronavigation becomes particularly valuable to compensate for the absence of real-time cortical testing. Thus, while awake mapping remains the optimal strategy for functional preservation, an individualised, multimodal approach ensures that non-candidates still benefit from safe and functionally guided resection. Nonetheless, ongoing research in neurocognitive mapping is developing new paradigms to test memory (e.g., recalling words or story details during DES), calculation, or even musical ability [45]. The philosophy of “cognitive neuro-oncology surgery” is to individualize the surgical plan to each patient’s brain and life goals, achieving maximal tumour removal while preserving the specific functions that define that patient’s identity and daily life. This approach, advocated by Duffau and others, represents a shift toward “connectomic neurosurgery” that prioritizes long-term quality of life as well as survival [9,49,50].

Awake craniotomy, though highly effective for preserving motor, language, and cognitive functions, can be psychologically demanding. Pre-existing anxiety, fatigue, or inadequate preparation may lead to intraoperative distress, agitation, or, rarely, termination of mapping. Careful patient selection, preoperative psychological counselling, and the involvement of an experienced neuropsychological team are critical to minimise emotional burden and ensure cooperation throughout the procedure [42,47,49]. Such counselling is critical, and has been proven effective; in another publication by the senior author, for example, an awake throughout craniotomy paradigm was used (where patients are awake through must of the procedure and only lightly sedated in specific portions); 92.3% of patients reported a willingness to repeat ATC in the future if necessary and minimal or no pain was reported in more than 80% of participants [43].

### 3.2. Intraoperative Magnetic Resonance Imaging (iMRI)

Even with the best preoperative planning and intraoperative functional mapping, a surgeon’s ability to achieve gross total resection can be hindered by the brain shifting during surgery and by limitations of the operating microscope as small tumour remnants can be hard to visually distinguish from normal brain [52]. Intraoperative MRI (iMRI) addresses this by providing real-time imaging updates during the resection. High-field iMRI suites (often 1.5T or 3T magnets) allow the surgeon to obtain a scan after an initial resection pass; if residual tumour is seen on the scan, the patient can be draped again and surgery resumed to remove the residual [53,54]. iMRI serves as an invaluable intraoperative checkpoint, enabling neurosurgeons to maximize the extent of resection by identifying its presence nearby important structures while improving patient survival and functional outcomes [53,54].

Numerous studies have confirmed that iMRI-guided surgery achieves a higher rate of complete resection compared to conventional surgery with navigation alone [55]. A meta-analysis of three randomized trials and multiple observational studies found that the use of iMRI increased gross total resection rates by an approximate factor of 1.4–1.6, including in low-grade gliomas where standard techniques often yield subtotal resection [54,56,57]. In that analysis, iMRI improved the percentage of tumour volume resected by an average of 5–7% and significantly boosted the odds of achieving 100% resection in both low-grade and high-grade gliomas [57]. Importantly, the meta-analysis noted no increase in neurological deficits or other adverse outcomes with iMRI use, indicating that the technique can safely extend resections [57]. Furthermore, some studies have found iMRI-associated resections translate into longer progression-free survival in malignant gliomas, although evidence for overall survival benefit is mixed (possibly confounded by subsequent therapies) [56,57]. However, iMRI also poses practical challenges due to prolonged operative time, increased anaesthetic exposure, and the strict sterile workflow required when moving between scanning and surgical phases. These factors can marginally elevate infection risk and operative fatigue [52,53,54,55,56,57]. Furthermore, resource intensity and limited availability restrict its universal adoption. Consequently, although techniques such as awake mapping and iMRI are indispensable in modern neuro-oncology, their use should be tailored to patient suitability, institutional capability, and multidisciplinary support. Ongoing improvements in workflow efficiency, perioperative anxiety management, and infection control are necessary to balance technological sophistication with patient safety.

The latest advances in iMRI include integration with surgical navigation and automated image analysis. Diffusion-weighted iMRI can check for acute ischemia after resection, and newer sequences can differentiate tumor vs. edema in the OR [52,55]. Some centres employ multiple iMRI scans (e.g., mid-resection and end) to guide stepwise resection. High-field 3T iMRI provides better image quality and smaller remnant detection than older low-field systems [58,59]. There is also interest in ultrahigh field (7T) iMRI, though its adoption is limited by cost and safety considerations. A recent randomized trial at 3T confirmed that high-field iMRI significantly improved extent of resection (EOR) and gross total resection (GTR) rates in both enhancing and non-enhancing tumour portions, particularly for low-grade gliomas (where defining tumour borders is challenging) [59,60,61]. However, it is important to note that iMRI is resource-intensive: it requires a specialized OR, longer operative time for scanning, and strict safety protocols and materials; for these reasons, not all centres have iMRI access [56]. Current best practice in many expert centres is to use iMRI for high-grade gliomas where achieving maximal resection correlates strongly with survival, and for select low-grade gliomas in critical areas to maximize safe removal [55]. In lower-resourced settings or those without iMRI, other adjuncts can in part substitute (e.g., ultrasound and fluorescence). Indeed, a consensus in the field is that while iMRI is extremely useful, it should be combined with other modalities for optimal results (such as, functional mapping to avoid deficits, and fluorescence to visualize the tumour) [60,62]. A review by Suero Molina et al. noted that combining 5-ALA fluorescence with iMRI achieved approximately 90% complete resection rates in high-grade gliomas when mapping was also used, shifting the cause of any incomplete resection from missed tumour to functional constraints (eloquence [62]. In other words, with multimodal guidance, surgeons can remove all tumour that is removable without harming function.

### 3.3. Intraoperative Ultrasound (iUS)

Intraoperative ultrasound has seen a resurgence as an attractive real-time imaging tool during brain tumour surgery. Modern high-frequency ultrasound probes provide improved resolution, and neuronavigational systems can now fuse ultrasound with preoperative MRI for easier interpretation (so-called navigated 3D ultrasound) [63]. The major advantages of iUS are its real-time feedback, portability, and low cost. Surgeons can use ultrasound throughout the resection to visualize the tumour and its boundaries, even as the brain shifts (overcoming a key limitation of static pre-op MRI) [64]. Ultrasound shows residual tumour as hyperechoic (bright) areas in many cases, enabling the surgeon to actively aspirate those areas and immediately see the effect on the image. It is also useful for detecting complications like intracerebral hematomas or excessive swelling during surgery, which can then be addressed promptly [63,65].

Recent advances in ultrasound technology have enhanced its utility in glioma surgery. Contrast-enhanced ultrasound (CEUS) involves injecting microbubble contrast agents during surgery; tumour tissue, being highly vascular or having a disrupted blood–brain barrier, often shows contrast enhancement on ultrasound [66,67]. Certainly, CEUS has demonstrated high sensitivity (90%) and specificity (99%), enabling precise differentiation of malignant lesions, intraoperative detection of glial tumours, and characterization of GBMs; it can thereby support surgical planning and enhance tumour resection by highlighting remnants that might blend in on standard B-mode ultrasound [65]. Another development is ultrasound elastography, which measures tissue stiffness. Tumours often have different elasticity than normal parenchyma, so elastography can delineate tumour margins by stiffness contrast [63,66,67]. Additionally, 3D ultrasound now allows the acquisition of a volume ultrasound dataset that can be navigated in multiple planes, resembling an MRI. This 3DUS can be updated repeatedly and fused with the neuronavigational coordinate system, mitigating brain shift issues [64,66,68]. Furthermore, research is also ongoing into ultrasound-based brain shift correction, using intraoperative US to adjust the navigation system’s MRI data after brain shift has occurred [69].

In terms of outcomes, several studies indicate that iUS contributes to maximizing resection. A systematic review by Wu et al. (2024) concluded that iUS usage is associated with higher resection rates and helps preserve function by providing continuous guidance [61,66]. Importantly, ultrasound can be used even in resource-limited settings where MRI is not available, increasing its global impact [64,68]. There are, however, pitfalls to be aware of: ultrasound image quality can be affected by operator experience and factors like acoustic artifacts, attenuation of deep structures, or blood in the field [64,68]. The learning curve is significant, initially, distinguishing tumour from normal tissue on ultrasound can be challenging. In high-grade gliomas, the tumour is often clearly delineated on US, but in low-grade gliomas (which may be iso-echoic) it can be subtle [65,66,67]. Techniques like slow systematic scanning, comparing with pre-operative images, and using Doppler/contrast help mitigate these issues [65,66,67]. One comparative study found that combining iUS with 5-ALA fluorescence achieved better resection rates than 5-ALA alone, suggesting that ultrasound can pick up non-fluorescing tumour portions or deeper extensions [70]. On the other hand, another study found no significant difference between 5-ALA alone versus 5-ALA plus intraoperative CT in high-grade gliomas, emphasizing that not all adjuncts add value; however, that study did not examine ultrasound [71]. The community consensus is that ultrasound is a highly useful adjunct for real-time resection control. Ongoing innovations, like better probe designs, ultrasound-integrated microscopes, and AI-driven ultrasound image analysis, promise to further enhance its effectiveness [63,64,66]. Given its cost-effectiveness and immediacy, iUS has solidified its role as a complement (or even alternative) to iMRI in centres without access to intraoperative MR scanning [63,64,66].

### 3.4. Intraoperative CT

Portable intraoperative CT (iCT) scanners (e.g., mounted on movable gantries or the O-arm system) can provide intraoperative imaging as well. In practice, iCT is more commonly used in spinal surgery or for quick checks (such as verifying catheter placement or detecting haemorrhage) rather than for guiding tumour resection, because its soft-tissue contrast is inferior to MRI and even ultrasound for parenchymal tumours [72,73,74,75]. In brain tumour surgery, iCT can depict gross features like midline shift or large haemorrhages, and can confirm that no sizable tumour piece was left, but it often cannot clearly differentiate residual infiltrative tumour from normal brain since both have similar X-ray attenuation [71,74]. A 2016 study examined the use of portable iCT combined with 5-ALA in high-grade glioma surgery. It reported that, while feasible, adding iCT did not significantly increase the extent of resection compared to using 5-ALA alone; presumably because 5-ALA already provided visual cue of residual tumour and CT did not further refine the margin [71]. Thus, iCT’s impact on EOR appears limited. Nevertheless, iCT can be useful for specific cases: for example, if an MRI-visible tumour nodule lies near the skull base bony structures, a CT scan can help confirm its removal or help navigate calcified regions [72,74]. iCT is also fast, acquiring a scan takes only minutes and does not require the lengthy safety preparations of iMRI [76]. In emergency situations or when MRI is contraindicated (e.g., certain implanted devices or if the patient is not stable), iCT might provide a quick intraoperative snapshot [76].

Current usage of iCT in glioma surgery is fairly limited, often reserved for adjunct purposes like confirming no acute complication or for stereotactic navigation updates when MRI is unavailable. As imaging moves more towards MRI and ultrasound, iCT has taken a backseat. However, new developments such as cone-beam CT with improved contrast or combined CT-fluoroscopy systems could potentially play a future role [77]. For instance, intraoperative CT angiography can visualize vascular anatomy if vascular involvement is a concern. In general, iCT remains an available tool, but compared to MRI and ultrasound, it has lower utility for soft tissue differentiation and thus contributes less to maximizing resection extent in gliomas [54,64,71].

### 3.5. Fluorescence-Guided Surgery (FGS) with Tumour-Targeted Dyes

One of the most notable intraoperative advances for gliomas has been fluorescence-guided surgery using tumour-selective fluorophores. The two main agents in use are 5-aminolevulinic acid (5-ALA) and sodium fluorescein, each of which causes tumour tissue to fluoresce under a specific light, helping surgeons visually discern tumour from normal brain in real time, thereby significantly improving glioma extent of resection [78,79,80].

5-ALA (Gliolan) is an orally administered prodrug that induces selective accumulation of the fluorescent metabolite protoporphyrin IX (PpIX) in tumour cells [80,81]. Under violet-blue illumination (≈400 nm) through a modified surgical microscope, high grade glioma tissue emits a red-violet fluorescence [80]. The landmark randomized controlled trial by Stummer et al. (2006), established 5-ALA as an effective adjunct for malignant gliomas: 5-ALA guidance more than doubled the rate of complete resection of contrast-enhancing tumour (65% vs. 36% with white-light alone) and improved 6-month progression-free survival [14]. This level I evidence led to widespread adoption of 5-ALA in Europe (approved in 2007) and more recently in the US (FDA approved in 2017 for high-grade glioma resection) [81]. The practical impact is significant; under blue light, residual tumour cells in the resection cavity often glow, alerting the surgeon to areas that should be further resected [80,81]. Surgeons report that fluorescence frequently extends beyond the MRI enhancement margin, highlighting infiltrative tumour that might otherwise be left behind. Indeed, 5-ALA-induced fluorescence can sometimes be seen in tissue that is non-enhancing on preoperative MRI; roughly 20% of non-contrast-enhancing gliomas (including some lower-grade tumours) exhibit areas of fluorescence, indicating foci of higher-grade tumour or metabolically active tumour cells [80,82,83,84]. The positive predictive value of visible fluorescence for tumour is very high (>95% in one series); if it fluoresces, it is almost certainly tumour tissue. This gives the surgeon great confidence in resecting fluorescent areas (assuming functional mapping indicates it is safe) [80,82,83,84].

Contemporary best practice with 5-ALA involves a multi-modal workflow that includes the use of the microscope’s fluorescence mode intermittently during resection to check for residual tumour, especially at the resection borders [14,85,86]. Many centres toggle between white-light and blue-light throughout the procedure; a continuous alternation between two visual modalities: blue-light, employed to detect fluorescent tumour tissue, whereas white-light provides the necessary resolution for precise anatomical dissection. This iterative process can be encapsulated in the guiding principle: “assess under blue, resect under white” [85]. The combination of 5-ALA with intraoperative imaging is synergistic. For example, if iMRI shows a small nodule of residual tumour, switching to blue light can help locate it in the cavity if it fluoresces. Conversely, if no fluorescence is seen but MRI suggests remaining enhancement, that could indicate non-fluorescing tumour, guiding further action [62,82,87]. Studies combining 5-ALA and iMRI have demonstrated some cases where iMRI prompted a search in an area that initially showed no obvious fluorescence, but upon re-inspection under blue light, additional fluorescent tumour was found and resected. This underscores that while 5-ALA is extremely useful, not all tumour cells will fluoresce (e.g., low-density infiltrating cells may be below visual threshold), so other adjuncts remain valuable [61,82].

It should be noted that 5-ALA is most effective for high-grade gliomas (WHO grade 3–4). Low-grade gliomas usually do not produce sufficient PpIX to be seen by the naked eye, although high-grade transformation areas within them might [14,78,82,83,87]. Research is underway on tools like hand-held spectrometry to detect lower levels of PpIX fluorescence that are not visible, potentially expanding 5-ALA use to lower grades. As of now, for diffuse low-grade gliomas, other methods (like mapping and iMRI) are the main adjuncts [14,78,82,83,87].

Sodium fluorescein is an intravenously administered fluorescent dye that exhibits high plasma protein binding. This property facilitates its accumulation in areas with a disrupted blood–brain barrier; thereby optimizing intraoperative visualization of high-grade gliomas and other enhancing tumour areas (neoplastic areas) which will preferentially take up fluorescein [81,88,89]. Under a surgical microscope equipped with a yellow 560 nm filter, fluorescein fluoresces bright yellow-green, highlighting tumour tissue [90]. Fluorescein has gained popularity as a more affordable, easy-to-use alternative to 5-ALA (no special patient ingestion hours before, just an IV injection during surgery) [90]. Several studies and a multicentre trial have demonstrated that fluorescein-guided resection is safe and effective for high-grade gliomas [88,91]. In the FLUOGLIO phase II study, 82.6% of patients achieved complete resection of enhancing tumour using fluorescein guidance, a rate comparable to that seen with 5-ALA [88,91]. Fluorescein’s visibility is excellent for superficial tumour; deeply seated tumours or those behind intact blood–brain barrier may not show fluorescence until resected to a point where contrast leakage occurs [88,91]. One recent comparative study found that fluorescein-guided resection significantly improved EOR and even prolonged overall survival in newly diagnosed glioblastomas compared to conventional white-light surgery [92]. Another report noted that fluorescein-guided surgery in eloquent or deep-seated HGGs enabled more extensive resection while preserving neurological function [93]. Thus, fluorescein is establishing its role, often used in centres where 5-ALA is not readily available or even in combination (some surgeons use 5-ALA for the main resection and fluorescein at the end to visualize any residual in a different way; though “double fluorescence” approaches are still being studied and validated) [88,91,94].

Overall, fluorescence-guided surgery has become a standard adjunct for malignant gliomas, recommended by neuro-oncology guidelines to maximize resection [14,88]. Key best practices include using the proper filters, timing the dye administration for optimal tumour uptake (e.g., fluorescein is often given after induction so that by the time of resection it is well-distributed), and being aware of the dye’s distribution (fluorescein highlights blood–brain barrier breakdown, so it may also brighten areas of radiation necrosis or inflammation; 5-ALA is more tumour-cell specific via metabolic uptake) [14,88]. Both dyes are very safe at the usual dosage, with minimal side effects (transient liver enzyme elevation for 5-ALA may occur in rare cases; yellowing of skin/urine with fluorescein can occur temporarily) [78,81,89]. Moreover, the success of these agents has led to the development of new targeted fluorophores; for example, compounds that bind specifically to EGFRvIII or other tumour markers, and fluorescent nanoprobes [14,88]. One such agent, tozuleristide (Tumor Paint BLZ-100, a chlorotoxin-based dye), has shown the ability to label tumour during surgery, though it is still under development [79,81,95,96]. Furthermore, Raman spectroscopy and desorption electrospray mass spectrometry (DESI-MS) are also additional emerging tools that can detect residual tumour at a molecular level in situ, potentially complementing visual fluorescence [97]. These innovations are part of a broader trend of molecular-guided surgery, aiming for ultra-precise differentiation of tumour from brain.

Table 2 provides a summary of intraoperative techniques used in glioma surgery.

## 4. Current Best Practices, Challenges, and Emerging Directions

Modern glioma surgery in eloquent brain regions typically employs a multimodal strategy. A paradigmatic scenario might include a preoperative high-resolution MRI with tractography and task/rest fMRI to map key functions and pathways [98]; followed by the surgical team formulating a plan, sometimes rehearsing on tractography-integrated neuro-navigation or using virtual reality to visualize the tumour and networks [15,40]. In the OR, neuronavigation is used for the craniotomy planning and initial tumour approach, taking into account the information obtained in the pre-operative imaging maps that show the functional zones [72]. If the tumour is near motor/language areas, an awake craniotomy with DES mapping is performed to function-test the boundaries of resection [13,41,45]. Intraoperatively, adjuncts like 5-ALA or fluorescein are employed to visually enhance tumour margins, and ultrasound or iMRI are used to detect any residual tumour that is not apparent on visual inspection [59,83]. This combination leverages the strengths of each tool; functional mapping prevents neurological injury, while imaging and fluorescence prevent leaving tumour behind and improving the extend of resection (and therefore overall survival and/or progression free survival) [99]. It is important to note that the integration and purpose of functional imaging and intraoperative mapping techniques differ between low-grade gliomas (LGG) and high-grade gliomas (HGG) due to their distinct biological behaviour and interaction with brain networks. LGGs, characterised by slow growth and high neuroplastic potential, frequently permit extensive cortical and subcortical reorganisation. In these patients, the surgical strategy prioritises maximal safe resection through fine-grained functional mapping, often using awake craniotomy and tailored cognitive tasks to exploit this plasticity and achieve supratotal resection when feasible [9,13,42,49]. Preoperative modalities such as task-based and resting-state fMRI, DTI, and connectome modelling are particularly valuable in LGG, as they reveal compensatory network recruitment and guide dynamic resection boundaries. Conversely, HGGs (including glioblastomas) exhibit infiltrative, rapidly progressive, and biologically aggressive behaviour with limited capacity for functional compensation. Here, the objective shifts from mapping-driven supratotal excision to maximising oncological resection within safely defined anatomical limits [52,53,54,55,56,57]. Technologies such as intraoperative MRI (iMRI) and 5-ALA fluorescence play a more prominent role in this context, facilitating precise delineation of tumour margins and minimising residual disease. Functional mapping remains essential in eloquent areas but is complemented by adjuncts like neuronavigation and intraoperative monitoring under general anaesthesia when time or tumour burden precludes awake mapping. Thus, while the philosophy of functional preservation unites LGG and HGG surgery, the degree of reliance on neuroplasticity-driven mapping versus imaging-guided radicality is determined by the tumour’s grade, growth kinetics, and the patient’s functional reserve and post-surgical functional objectives.

Despite these significant advances, challenges remain. One ongoing challenge is the integration and interpretation of multimodal data. Surgeons must synthesize information from fMRI, DTI, intraoperative mapping, and imaging, which can sometimes be conflicting (e.g., fMRI suggests an area is non-functional, but DES finds it is essential; DES is usually trusted more) [45]. Furthermore, there is a learning curve to each technology, and even in expert hands, limitations like false negatives (e.g., a functional area that neither fMRI nor DES identified) can occur [45]. Neuroplasticity adds complexity as brain networks can rewire around slow-growing tumours, meaning function might relocate. Preoperative mapping only captures one time-point; repeated mapping postoperatively and during tumour recurrences might be needed (an emerging concept of longitudinal mapping and repeat resections over years) [100,101]. Although significant progress has been made in integrating advanced neuroimaging and mapping modalities into brain tumour surgery, it is essential to critically assess the maturity of each technology and the robustness of supporting evidence. Some modalities, such as task-based fMRI, DTI tractography, and direct electrical stimulation (DES), are supported by extensive validation and have demonstrable impact on surgical outcomes [11,18,25,26,30,33]. By contrast, resting-state fMRI (rs-fMRI), despite growing clinical adoption, still faces challenges. While rs-fMRI can localise language networks and may be particularly helpful in patients unable to perform tasks [19,22,23,24], its clinical reliability remains limited by variable preprocessing pipelines, lack of standardised thresholds, and susceptibility to motion and physiological noise [20,21,22]. Correlation between rs-fMRI maps and intraoperative stimulation results can be inconsistent, particularly for higher-order association networks such as the DMN, SN, and ECN [20,21,23]. Furthermore, emerging connectomic approaches and predictive network models show promise, but many remain supported by early-stage, single-centre cohorts without external validation [37,38,39]. Large-scale, harmonised, multicentre studies are therefore needed to establish reproducibility, define clinically meaningful effect sizes, and determine cost-effectiveness before these technologies can be fully integrated into routine surgical practice.

While our review focuses primarily on the neuroimaging and intraoperative mapping techniques that enable the preservation of cognitive networks, it is important to acknowledge that the ultimate validation of “functional preservation” lies in neuropsychological outcomes. Standardised assessment tools and follow-up protocols, however, remain highly heterogeneous across studies. The timing of cognitive testing varies widely, ranging from early postoperative evaluations to long-term longitudinal assessments, making meta-analytic synthesis difficult. Moreover, different centres employ diverse batteries, such as the Addenbrooke’s Cognitive Examination (ACE), Montreal Cognitive Assessment (MoCA), or Comprehensive Neuropsychological Assessment in Oncology (CNAO), often with adaptations that preclude direct comparison. Given this variability, the present review does not attempt to systematically appraise psychometric methods or outcome measures, as doing so would require a separate methodological framework. Nonetheless, future work should aim to integrate structured cognitive testing at predefined time-points (for example, preoperatively, at three months, and at one year post-surgery) to establish unified benchmarks of cognitive preservation and facilitate cross-centre comparability. Linking these neuropsychological data with connectome-based imaging findings would further strengthen the objective definition of functional preservation.

Another challenge is resource availability. Not every hospital can afford an iMRI suite or even the consumables for 5-ALA. An iMRI suite entails substantial investment in high-field MRI systems, shielded operating rooms, and trained technical staff, often exceeding one million USD in setup costs and significantly increasing operative time. Similarly, 5-ALA, while highly effective for delineating high-grade gliomas, involves recurring expenses for photosensitiser agents and specialised blue-light microscopes. In contrast, intraoperative ultrasound (iUS) offers a cost-effective and immediately available alternative: it enables real-time resection control, is portable, and requires only moderate training, making it suitable for resource-limited settings. Evidence indicates that combining iUS with 5-ALA can achieve resection rates comparable to iMRI-guided surgery, underscoring its potential as a scalable substitute [64,65,66,67,68,69,70]. To enhance global equity in neuro-oncological care, a tiered technological model could be helpful; in high-income settings, multimodal integration of iMRI, 5-ALA, and connectomic guidance remains ideal for maximising resection while preserving function. In middle-income regions, a pragmatic combination of iUS, neuronavigation, and low-cost fluorescence microscopy (such as fluorescein-guided surgery) can replicate much of this benefit, while in low-resource environments, capacity building through training in navigated 3D ultrasound, integration of preoperative MRI data, and tele-mentoring initiatives could substantially elevate surgical precision at minimal cost. Certainly, there is a need for cost-effective solutions like widely sharing ultrasound expertise and perhaps using lower-cost innovations (e.g., augmented reality microscopes) that overlay tractography or fMRI data [61,63]. Augmented reality (AR) and neuronavigational improvements are indeed on the horizon, some systems now project functional MRI activation maps or diffusion tractography directly in the eyepiece of the microscope, so the surgeon “sees” the invisible functional boundaries during resection [102]. Similarly, robotic assistive devices are being explored to enhance precision; for instance, robots could potentially be guided by the surgeon’s preoperative plan and intraoperative updates to ensure trajectory constraints are respected [103]. These and other strategies align with the World Health Organization’s call for context-appropriate surgical innovation (https://www.who.int/publications/i/item/9789240095212, accessed on 15 October 2025) and highlight the need for economic analyses comparing these modalities’ incremental cost-effectiveness ratios to clinical outcomes. Ultimately, adapting technological sophistication to local contexts, rather than transplanting resource-intensive models, represents the most sustainable pathway toward equitable functional neurosurgery and an important avenue to further validate and study.

Emerging technologies that could further transform the field include ultrahigh field 7-Tesla MRI (both preoperative and possibly intraoperative in the future), which offers unprecedented resolution to delineate tumour from functional tissue. The higher signal-to-noise at 7T has shown the ability to map smaller cortical columns and detect subtle activations that 3T might miss [104]. This could be especially useful in mapping finer cognitive functions or in small brain structures (like memory-related structures in the medial temporal lobe) [104]. Artificial intelligence (AI) can also be of aid in the future, machine learning algorithms can assist in image segmentation (to highlight tumour vs. oedematous brain), denoise fMRI data, or even predict functional outcomes based on a patient’s connectome and proposed resection [105]. AI tools are being developed to analyse intraoperative ultrasound or to provide “smart” suggestions during surgery (like warning the surgeon if they are approaching a critical fibre tract, based on real-time tool tracking and pre-operative data) [105,106,107]. Another frontier is real-time intraoperative metabolic analysis, where devices like DESI-MS can chemically analyse resection cavity margins in seconds, telling the surgeon if residual tumour metabolites are present [108]. Similarly, confocal endomicroscopy probes can give a microscopic fluorescent view of cells in situ, potentially identifying single-cell infiltrations at the margin [109]. However, significant technical and interpretive bottlenecks remain before these tools can be adopted in routine clinical practice. In the case of AI, model performance is often constrained by the limited size, heterogeneity, and imbalance of available training datasets, which are rarely representative of the full spectrum of glioma subtypes and surgical contexts [105,106,107]. Furthermore, the black-box nature of many deep learning algorithms limits interpretability, impeding clinical trust and regulatory approval [105,106,107]. Explainable AI frameworks and harmonised, multicentre datasets are therefore needed to achieve reproducible and ethically sound applications. Similarly, 7-Tesla MRI offers unparalleled spatial resolution for delineating tumour infiltration and cortical microarchitecture, yet it is affected by pronounced susceptibility artefacts, geometric distortions, and specific absorption rate constraints that can degrade image quality near air–tissue interfaces and limit patient safety [104,105]. Hardware and sequence optimisation, along with automated distortion-correction pipelines, are essential before 7T imaging can be routinely integrated into operative workflows. Finally, molecular imaging techniques, such as amino-acid PET or hybrid PET/MRI, hold promise for visualising tumour metabolism and distinguishing recurrence from treatment effects, but they remain limited by tracer availability, high costs, and the absence of universally validated quantitative thresholds. Addressing these limitations through multicentre standardisation and validation initiatives will be necessary for translating these technologies from experimental research into reliable tools for connectome-guided neurosurgery.

The ultimate measure of success is patients who live longer and better (i.e., extended survival without neurological disability). On that front, the trend is encouraging. For low-grade gliomas, maximal safe resection has extended median survival from approximately 7 years (with minimal resection) to well over 15 years in recent reports where an aggressive, mapping-guided approach was taken [110]. Quality of life assessments in glioma patients who underwent awake cognitive mapping show that many can return to work and normal activities, with high rates of preservation of language, memory, and executive function [9,43,50]. For glioblastomas, although the disease remains fatal, more complete resections (combined with adjuvant therapy) have been shown to improve survival by a few months to a year, which is non-trivial in this context [4,111]. Moreover, with careful functional monitoring, the proportion of patients with severe postoperative deficits has dropped substantially at high-volume centres, despite resections being more aggressive than in the past [112].

## 5. Conclusions

Modern brain tumour surgery is entering an era defined by functional preservation within oncological radicality, driven by the convergence of connectomics, intraoperative mapping, and advanced imaging. However, despite substantial technological progress, several important scientific challenges remain unresolved. First, there is a pressing need to validate and standardise multimodal neuroimaging protocols, including task-based and resting-state fMRI, diffusion models, and connectomic reconstructions across centres to ensure reproducibility and clinical reliability. Second, integration of imaging with longitudinal neuropsychological outcomes must become routine, establishing objective metrics of cognitive preservation and recovery. Third, future work should focus on quantitative connectome-based predictive models that can forecast postoperative deficits, guide rehabilitation, and individualise risk-benefit decisions. Additionally, interpretability and transparency in AI-driven analytics and biophysical limitations in ultrahigh-field MRI must be addressed through collaborative benchmarking and data-sharing initiatives. Finally, achieving global equity in functional neurosurgery requires context-appropriate, cost-effective technological frameworks for resource-limited settings. In general, the next phase of progress depends less on novel devices and more on integrating multimodal evidence, neuropsychological validation, and international collaboration to transform functional preservation from an empirical goal into a reproducible, patient-centred one.

## Figures and Tables

**Table 1:** Comparison of Core MRI-Based Techniques in Brain Tumour Mapping and Connectomes.

Modality	Principle	Key Advantages	Main Limitations	Practical Impact
Functional MRI (fMRI)	Measures blood-oxygen-level-dependent (BOLD) signal changes associated with neural activation during specific cognitive or motor tasks.	Non-invasive, high spatial resolution, enables mapping of eloquent cortices preoperatively.	Prone to false positives and negatives in peritumoural regions due to altered neurovascular coupling; results depend on task performance and patient compliance.	Useful for preoperative localisation of eloquent areas, but intraoperative confirmation using direct cortical stimulation (DCS) remains the gold standard.
Resting-State fMRI (rs-fMRI)	Analyses spontaneous BOLD signal fluctuations to infer functional connectivity without active tasks.	Suitable for patients unable to perform tasks; reveals intrinsic network organisation and compensatory plasticity.	Reduced specificity compared with task-based paradigms; sensitive to motion artefacts and haemodynamic disturbances in tumoural tissue.	Provides complementary information on network-level reorganisation and helps guide functional preservation strategies.
Diffusion Tensor Imaging (DTI)	Models anisotropic water diffusion to estimate the orientation and integrity of white matter tracts.	Allows visualisation of major white matter pathways and planning of safe resection corridors.	Distortions near tumours due to oedema, infiltration, and crossing fibres; limited accuracy in complex fibre regions.	Informs surgical approach to preserve critical tracts (e.g., corticospinal, arcuate fasciculus), though integration with intraoperative mapping is essential.
Advanced Diffusion Models (HARDI, CSD, DSI)	Employ higher-order diffusion models to resolve crossing or complex fibre geometries.	Greater tractography accuracy in infiltrated or oedematous tissue; improved delineation of associative pathways.	Requires long acquisition times and advanced post-processing; still influenced by tumour-related signal alterations.	Enhances the reliability of connectome-based surgical planning and patient-specific mapping of eloquent networks.

**Table 2:** Comparative overview of intraoperative imaging and fluorescence-guided modalities used in glioma surgery.

Modality	Mechanism/Principle	Main Advantages	Limitations	Impact on EOR and Functional Outcome	Representative Evidence
**Intraoperative MRI (iMRI)**	Real-time magnetic resonance scanning (1.5T–3T) during surgery allows updating of neuronavigation and detection of residual tumour after partial resection.	High-resolution anatomical imaging; compensates for brain shift; identifies residual tumour; allows repeated scanning during procedure.	High cost and infrastructure requirements; longer operative time; limited availability.	Increases gross-total resection rates by ≈1.4–1.6× without increasing morbidity; improves progression-free survival in HGGs.	[55,57,59]
**Intraoperative Ultrasound (iUS)**	Real-time acoustic imaging (B-mode, contrast-enhanced, or 3D navigated) for visualising tumour boundaries and residual tissue.	Portable, inexpensive, immediate feedback; useful throughout resection; overcomes brain-shift limitations.	Operator-dependent; limited soft-tissue contrast in deep or iso-echoic tumours; learning curve.	Enhances intraoperative localisation and increases EOR, particularly when combined with fluorescence; reduces residual tumour volume.	[61,62,66]
**Intraoperative CT (iCT)**	Portable cone-beam or O-arm CT provides X-ray-based volumetric imaging during surgery.	Fast acquisition; useful for verifying resection cavity, haemorrhage, or bony involvement; compatible with neuronavigation.	Poor soft-tissue contrast compared with MRI/US; radiation exposure; limited discrimination of infiltrative tumour.	Marginal additive value for EOR in gliomas; mainly used for safety checks and stereotactic updates.	[14,60]
**Fluorescence-Guided Surgery (FGS)**	Tumour-specific fluorophores (5-ALA → PpIX; sodium fluorescein) visualised under filtered microscopy to highlight tumour tissue.	Real-time visual contrast; improves delineation of margins; inexpensive compared with iMRI; synergistic with other modalities.	Limited sensitivity in LGG; false positives in inflamed or necrotic tissue; requires specific microscope filters.	Doubles complete resection of enhancing tumour (65% vs. 36% with white light); improves 6-month PFS without higher morbidity.	[14,62,88,91]

Abbreviations: EOR, extent of resection; PFS, progression-free survival; HGG, high-grade glioma; LGG, low-grade glioma; 5-ALA, 5-aminolevulinic acid; PpIX, protoporphyrin IX.

## Data Availability

All data is available in the manuscript.
